# Pelvic organ prolapse and uterine preservation: a survey of female gynecologists (POP-UP survey)

**DOI:** 10.1186/s12905-020-01105-3

**Published:** 2020-10-27

**Authors:** Peter Urdzík, Vladimir Kalis, Mija Blaganje, Zdenek Rusavy, Martin Smazinka, Martin Havir, Rastislav Dudič, Khaled M. Ismail

**Affiliations:** 1 Department of Gynecology and Obstetrics, Faculty of Medicine, Safarik’s University and L. Pasteur Teaching Hospital in Kosice, SNP Street No. 1, 04001 Košice, Slovak Republic; 2 Biomedical Center, Faculty of Medicine in Pilsen, Charles University, alej Svobody 80, 304 60 Plzeň, Czech Republic; 3 Department of Obstetrics and Gynecology, University Hospital, Charles University, alej Svobody 80, 304 60 Plzeň, Czech Republic; 4 Division of Gynecology, University Medical Centre Ljubljana, Šlajmerjeva 3, 1525 Ljubljana, Slovenia; 5 Department of Gynecology and Obstetrics, Faculty of Medicine in Pilsen, Charles University, alej Svobody 80, 304 60 Plzeň, Czech Republic

**Keywords:** Preference, Survey, Attitude, Prolapse, Hysterectomy, Sparing surgery

## Abstract

**Background:**

The aim of this study was to explore the personal views of female gynecologists regarding the management of POP with a particular focus on the issue of uterine sparing surgery.

**Methods:**

A questionnaire based survey of practicing female gynecologists in the Czech Republic, Slovenia and Slovakia.

**Results:**

A total of 140 female gynecologists from 81 units responded to our questionnaire. The majority of respondents stated they would rely on a urogynecologist to aid them with their choice of POP management options. The most preferred options for POP management were sacrocolpopexy and physiotherapy. Almost 2/3 of respondents opted for a hysterectomy together with POP surgery, if they were menopausal, even if the anatomical outcome was similar to uterine sparing POP surgery. Moreover, 81.4% of respondents, who initially opted for a uterine sparing procedure, changed their mind if the anatomical success of POP surgery with concomitant hysterectomy was superior. Discussing uterine cancer risk in relation to other organs had a less significant impact on their choices.

**Conclusions:**

The majority of female gynecologists in our study opted for hysterectomy if they were postmenopausal at the time of POP surgery. However, variation in information provision had an impact on their choice.

## Background

One in 9 women undergo a form of reconstructive surgery for pelvic organ prolapse (POP) during the course of their life and this is expected to increase with the prolongation in life expectancy [1]. With improving operative safety and anesthetic techniques, such surgical procedures are more frequently performed on perimenopausal and postmenopausal women [2–5]. Indeed, in a study by Kalis et al. [6], 108 (89.3%) of 121 women undergoing laparoscopic sacrocolpopexy were > 50 years. Until recently, vaginal hysterectomy was the most common operation in POP management [7, 8]. The main proposed reasons for removal of the uterus were to obtain access to the supporting pelvic structures and/or to reduce the size of the prolapsed mass [9]. Nevertheless, the uterus itself seems to play only a passive role in the etiology of prolapse [10, 11] and therefore its removal without a thorough discussion with the patient, about the pros and cons of doing so, may be considered clinically substandard and unethical as it disregards the women’s autonomy and basic right of informed choice [12–14]. This issue is exacerbated by the paucity of long-term data on the psychological impact of hysterectomy on women [15].

Several studies [14, 16, 17] have explored women views about the issue of uterine preservation versus concomitant hysterectomy at the time of reconstruction procedures for POP. The heterogeneity in these studies’ findings is not unexpected given the impact of several factors including the individual’s values, cultural beliefs, level of education, ethnicity, age and family pressure [16, 18–20]. However, in these studies the target population did not specifically have prior medical knowledge hence their decision could have been biased not only by the information provision but rather by how they interpret such information. This is particularly relevant because, in essence, they are making a decision to remove a healthy organ based on projected assumed risks [14, 16, 21]. In order to mitigate the risk of such bias while ensuring that the woman’s perspective is taken into account, we decided to target a cohort of women with specialist knowledge in the field of gynecology in general and urogynecology in particular. In this study we undertook a survey of female gynecologists from different European countries with the aim of exploring their personal views about different aspects of management of POP. We also wanted to particularly focus on their choice of whether to preserve the uterus or not in response to different clinical scenarios.

## Methods

The study was undertaken between January and December of 2018 and involved 120 departments of Gynecology and Obstetrics located in the Czech Republic, Slovenia and Slovakia. A national coordinating center (NCC) located in each of the participating countries managed the study elements in that country. The NCCs were based at the Departments of Gynecology and Obstetrics Safarik’s University and L. Pasteur Teaching Hospital (SULPTH), University Hospital in Pilsen (UHP) and University Medical Centre Ljubljana (UMCL) in Slovakia, Czech Republic and Slovenia respectively. Anonymized questionnaires were sent by email from the lead investigator of each of the NCCs (UP, KV, BM) to the heads of departments of all the obstetrics and gynecology units in their relevant countries. They were asked to cascade them as hard copies to practitioners who fulfilled a set of a priori specifications. Completed questionnaires were returned by post to the relevant NCC for data extraction. The study received ethical approval from the relevant ethics committee at Ethics committee at L. Pasteur Teaching Hospital in Košice in Slovakia (No. 2020/EK/04024). While UHP in Czech Republic (waiver—April 30, 2020) and UMCL in Slovenia (waiver—April 22, 2018) committees waived ethical approval because of the nature of the study.

### Participants, inclusion and exclusion criteria

Participants were hospital-based female specialist obstetricians and gynecologists who have completed their postgraduate training and working as generalists or subspecialists in obstetrics and gynecology.

### Variables

We used a bespoke questionnaire based on previously published study exploring women’s perception of hysterectomy and attitudes towards uterine preservation at the time of POP reconstructive surgery [16]. The questionnaire was distributed in the native language of each of the participating countries (the questionnaires in the participating countries native languages are available on request, the questionnaire in English language—see in Additional file 1). Demographic details collected included participants’ age, country of residence, type of hospital they work at, sub-specialization or main area of special interest (materno-fetal medicine, oncogynecology, urogynecology, reproductive medicine, others, no sub-specialty). We also asked about future fertility plans using a 4-point Likert scale ranging from “not at all” to “I definitely will”.

Participants were asked to assume they are postmenopausal healthy women, with no prior major gynecological surgeries, suffering from a significant POP involving all compartments (i.e. anterior, apical, posterior), participants were then asked to respond to a set of questions and hypothetical scenarios to explore the following issues:

- Resources or people they would consult to aid them with the decision making about the best treatment for their POP.
- Their preferred type of management for their POP.
- The importance of the uterus to them.
- Potential factors that can affect their decision to have a hysterectomy.
- How important is anatomical outcome on their choice about concomitant hysterectomy with POP surgery.
- Would presenting life-long risk of uterine cancer in the context of other organ cancers impact their decision about choice of procedure?

### Statistical methods

Sample characteristics were summarized using descriptive statistics. Where relevant exact McNemar, Fischer tests and chi-squared test were performed using IBM SPSS Statistics 21.0 and Stata/SE 11.1 (StataCorp LP, College Station, TX, USA) statistical softwares. The cut-off for statistical significance was set at *p* < 0.05.

## Results

### Sample description

Of the 120 approached departments, questionnaires were returned from 81 (67.5%) of them. A total of 140 female gynecologists completed the questionnaire with a mean age of 38.7 years (range 28–67 years). Of these, 84 (60.0%), 31 (22.1%) and 25 (17.9%) were from the Czech Republic, Slovakia and Slovenia respectively. Participants were based at university or teaching hospitals (n = 82, 58.6%), regional hospitals (n = 39, 27.8%) and district hospitals (n = 19, 13.6%). All participants were fully specialized gynecologists, of these 23 (16.4%) were feto-maternal, 16 (11.4%) urogynecology and 4 (2.9%) oncogynecology subspecialists. With regard to future fertility plans, 49 participants (35.0%) stated that they completed their family while the remaining 91 (65.0%) either partially or not at all.

### Information provision

Based on the requested assumed scenario that participants were healthy, postmenopausal, with no prior gynecological surgeries and suffering with a significant POP involving all compartments, relying on a urogynecologist as a source of information was chosen by 130 (92.9%) of the participants as the main information resource. While searching the medical literature, consulting their partner or colleague was chosen by 54 (38.6%), 24 (17.1%) and 14 (10.0%) of the respondents respectively. Ten participants only have indicated that they would also seek assistance from online resources (n = 6, 4.3%), a female friend (n = 3, 2.1%) or an oncogynecologist (n = 1, 0.7%).

### Management preference

**Fig. 1:**
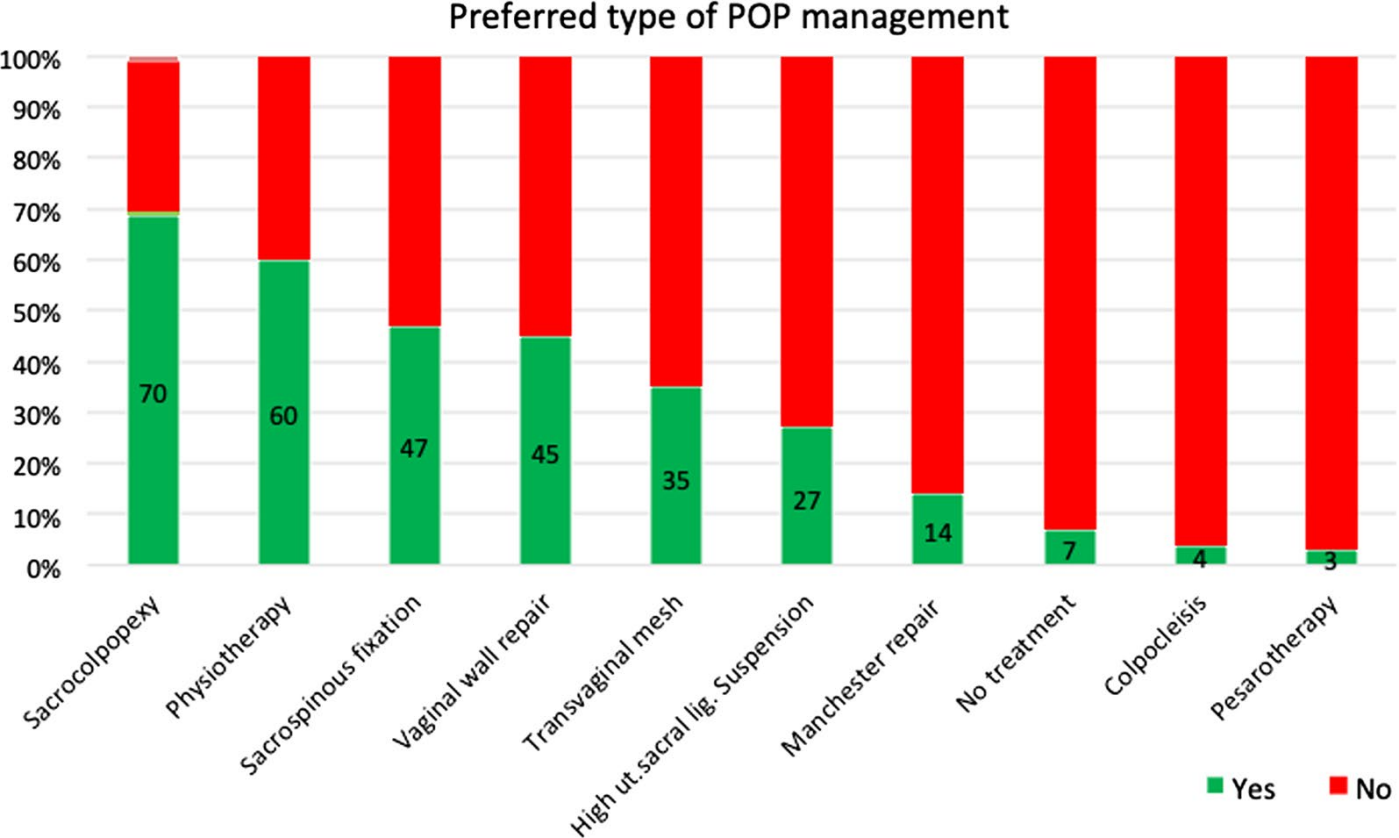
Personal management preferences for POP. Number of responders ranged from 126 to 134

Using the same assumption above, participants were asked to rate their likelihood of choosing different management options for POP on a 4-point Likert scale which was later dichotomized to “yes”, for definitely and likely, and “no”, for not likely and not at all (Fig. 1). The options favored by respondents, when combining definitely and likely responses, were sacrocolpopexy and physiotherapy. While a Manchester repair, no treatment, colpocleisis, and the use of a pessary were the least favored amongst female gynecologists.

### Factors impacting decision about hysterectomy

**Fig. 2:**
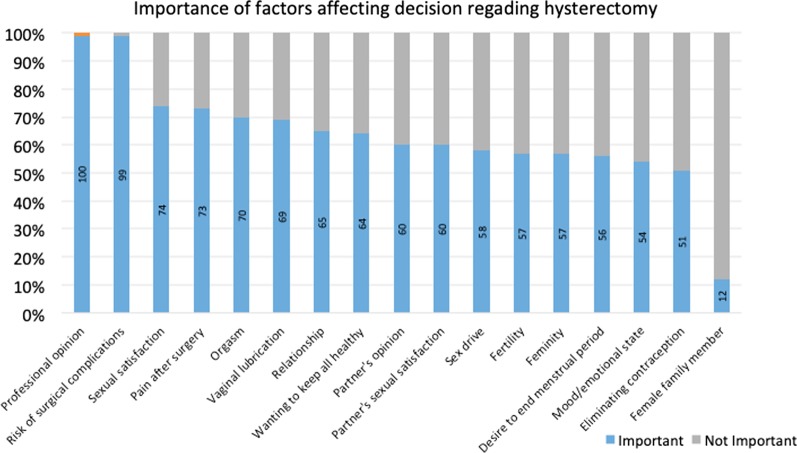
Importance of factors for decision to undergo or refuse hysterectomy. Number of responders ranged from 133 to 138

Participants were asked about their views regarding the importance of various factors on their decision to opt for or decline a hysterectomy, during POP reconstructive surgery, if both were feasible options. Professionals’ opinion and risk of surgical complications were considered important by 100% and 99% of respondents respectively. The list of factors assessed ranked in order of their importance based on participants’ responses are demonstrated in Fig. 2.

When asked about their personal perception about the importance of the uterus for their sense of self, 79/136 (58.1%) of respondents did not support this view. Of the 57 female gynecologists considering the uterus to be important for their sense of self, 33 (57.9%) said they would opt for a uterine sparing surgery than a hysterectomy compared to 18 of the 79 (22.8%) who did not support this view (OR = 4.66, *p* < 0.05).

### Impact of clinical outcome and risk of cancer on choice of surgery

**Fig. 3:**
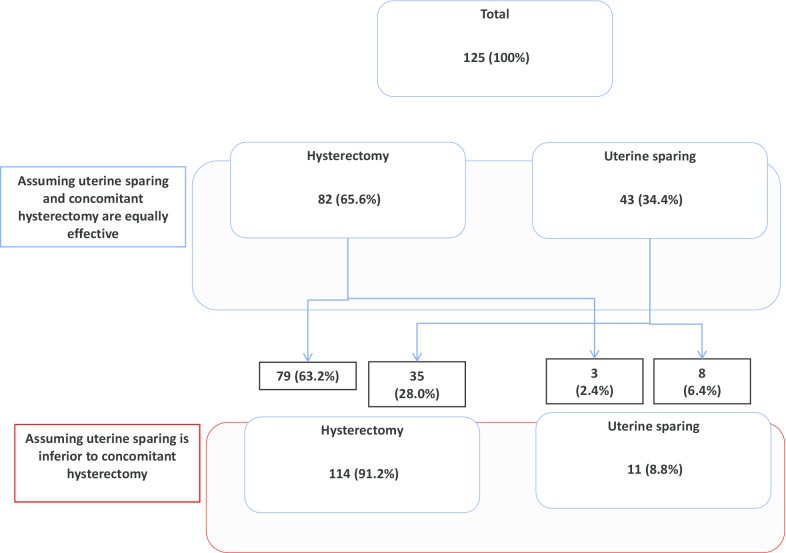
Differences in decision about POP management (hysterectomy vs. uterus sparing surgery) based on expected outcomes (n = 125)

When participants were asked about choice of surgery if there was evidence to suggest that anatomical outcomes following POP surgery with uterine sparing were similar to concomitant hysterectomy, 82/125 (65.6%) still opted for a concomitant hysterectomy. When asked about their choice if there was evidence that uterine sparing is associated with slightly worse outcomes, 35/43 (81.4%) who initially opted for uterine sparing changed their mind to a concomitant hysterectomy (Fig. 3).

**Fig. 4:**
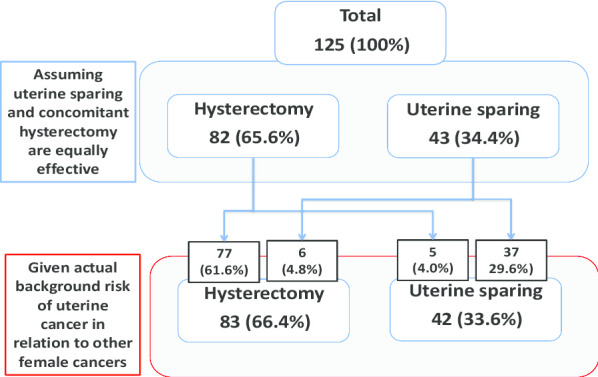
Differences in decision about POP management (hysterectomy vs. uterus sparing surgery) based on cancer risk (n = 125)

When information on actual background potential risk of uterine cancer in relation to other types of cancers in females was provided while still assuming equal effectiveness of uterine sparing and concomitant hysterectomy POP procedures, 5 (6.1%) women changed their decision from hysterectomy to uterus sparing surgery and 6 (13.9%) women from uterus sparing surgery to hysterectomy (Fig. 4). Additionally, 122 (87.1%) respondents stated that they would need to know the recent cervical screening result and 93 (66.4%) to have a transvaginal ultrasound assessment of their endometrial thickness preoperatively to enable them to make a well informed decision regarding hysterectomy or uterus sparing procedure. The choice of surgery depending on the different scenarios by country is presented in Table 1.

**Table 1:** Impact of clinical outcome and risk of cancer on choice of surgery by country

Scenario	Hysterectomy			Uterine preservation			*p*
	SK n = 31	CZ n = 84	SL n = 25	SK n = 31	CZ n = 84	SL n = 25	
If there was evidence to suggest that anatomical outcomes following POP surgery with uterine sparing were similar to concomitant hysterectomy	18 (58.1%)	52 (61.9%)	12 (48.0%)	13 (41.9%)	24 (28.6%)	6 (24.0%)	0.59
If there was evidence that uterine sparing is associated with slightly worse outcomes	26 (83.9%)	72 (85.7%)	16 (64.0%)	5 (16.1%)	4 (4.8%)	2 (8.0%)	0.19
Provided with information on the actual background potential risk of uterine cancer in relation to other types of cancers in females	20 (64.5%)	51 (60.7%)	12 (48.0%)	11 (35.4%)	25 (29.8%)	6 (24.0%)	0.96

*SK* Slovakia, *CZ* Czech Republic, *SL* Slovenia

*p* < 0.05 (Chí-squared test)

## Discussion

### Summary of results

This study presents personal views of female gynecologists on the issue of POP management with a particular focus on their choice of whether to preserve the uterus or not in response to different hypothetical clinical scenarios. The vast majority of our study participants stated they would rely on a urogynecologist as the main source of information to aid them with their choice of POP management options while, 4.3% and 0.7% only, would use online resources or seek the advice of an oncogynecologist, respectively, to help them make a decision.

The most preferred options for POP management were sacrocolpopexy and physiotherapy. Almost 2/3 of female gynecologists who responded to our questionnaire opted for a hysterectomy together with POP surgery, if they were menopausal, even if the anatomical outcome was similar to uterine sparing POP surgery. Moreover, 81.4% of respondents, who initially opted for a uterine sparing procedure, changed their mind if the anatomical success of POP surgery with concomitant hysterectomy was superior. Significantly more respondents changed their mind from uterine preservation to hysterectomy when asked to consider that uterine sparing might be associated with a slightly more negative clinical outcome compared to when asking them to consider their uterine cancer risk (8/43 vs. 35/43, *p* = 0.000).

### Comparison to current literature

The majority (65.6%) of female gynecologists would opt for hysterectomy if they were postmenopausal at the time of POP surgery, a proportion higher than that reported from previous women surveys whose participants were not recruited because of a particular professional background [16, 17].

Limited research exists that assesses patient knowledge of POP treatment options and attitudes regarding hysterectomy and its association with their perception of sexuality, femininity and womanhood [18, 22–24]. Nevertheless, it is the general opinion that the two crucial determinants of the patients’ choice of POP surgical technique are the woman’s personal views about uterine preservation and the surgeon’s procedure preference based on their training and expertise [25, 26]. It is interesting to see that the majority of specialist female gynecologists seem to prefer a hysterectomy even when quoted similar anatomical success or when highlighting the proximity in life-time uterine cancer risk to other organs. The impact of this issue is more relevant when considering that our respondents are clinicians who can be counseling patients rather than as a woman considering her own options.

Our findings concur with other groups, [14, 27, 28], where we demonstrated that the issues of the impact of a hysterectomy on femininity, sex drive and sexual satisfaction, either for the woman or her partner, did not seem to be a priority in the decision-making process regarding hysterectomy at time of POP surgery. However, the impact of hysterectomy on clinical outcomes seemed to be an important factor when choosing the optimal procedure. Similar to those of Korbly et al. [14] and van IJsselmuiden et al. [17] the number of women opting for uterine sparing surgery in our study significantly reduced if this was associated with slightly inferior anatomical outcomes (34.4–8.8%; OR 11.6, *p* < 0.001). A large RCT comparing uterine preservation surgery versus vaginal hysterectomy for POP repair reported similar anatomical and functional outcomes at 12-months [29]. In our survey sacrocolpopexy was the most preferred surgical procedure amongst female gynecologists. This is not surprising given the high-level evidence indicating that abdominal and laparoscopic sacrocolpopexy achieve better anatomical outcomes compared to other surgical options [30]. Several studies compared laparoscopic hysteropexy against total or subtotal hysterectomy with sacrocolpopexy with conflicting results [31–34]. Nonetheless, randomized comparisons of outcomes following sacrocolpopexy with or without uterine preservation are needed. Furthermore, it would also be prudent to explore alternative techniques and modifications that can overcome any variation in outcome [35]. This will ensure that women have two realistic and equal options to choose from. It is also important that women have access to specialized multidisciplinary expertise and validated instruments to help them evaluate their needs and hence make an individualized informed choice about their management [24].

### Strengths and limitations

We appreciate that our study has some limitations including the inability to know the exact number of specialists who received the questionnaire to be able to calculate an accurate response rate. Therefore, it is difficult to assess the risk of selection bias in this survey. However, the fact that our participants are specialized professionals working in different types of units from 3 different countries is reassuring that our sample is representative of the views of female gynecologists currently working in Central Europe. Moreover, the mean age of our participants was 38.7 years and several of them have not completed their families, yet they were asked to base their responses on the hypothetical assumption that they were postmenopausal. It could be argued that the views presented in this study might not be a true reflection of what postmenopausal female gynecologist would do. Nonetheless, it still reflects, to a large extent, what their perception is about the optimal modality of management for a postmenopausal healthy woman. This is of particular importance because of the potential impact this might have on others if we consider their roles as clinicians and trainers. In contrast, the fact that this is the first survey exploring views of female gynecologists about POP management and their preferences about uterine sparing or not is a major strength to our work.

## Conclusion

Concomitant hysterectomy rather than uterine sparing seems to be the preferred option for the majority of female gynecologists if they were to have POP reconstructive surgery. Urogynecologists were deemed the most important resource for our respondents when making a decision about the optimal management of their POP. Moreover, postoperative clinical outcome was an important determinant in their decision about the uterine fate. Therefore, there is an urgent need for information about the short and long-term clinical and patient-reported outcomes of uterine sparing versus concomitant hysterectomy POP surgery to enable women make an informed choice of the best management for them.

## Supplementary information

**Additional file 1**. Pelvic Organ Prolapse and Uterine Preservation: A survey of female gynecologists (POP-UP survey).

## Data Availability

The datasets used and/or analyzed during the current study are available from the corresponding author on reasonable request.

## Abbreviations

POP: Pelvic organ prolapse
NCC: National coordinating center
SULPTH: Safarik’s University and L. Pasteur Teaching Hospital
UHP: University Hospital in Pilsen
UMCL: University Medical Center Ljubljana
SK: Slovakia
CZ: Czech Republic
SL: Slovenia
