# Supplementary material for: Pelvic organ prolapse and uterine preservation: a survey of female gynecologists (POP-UP survey)
Source: BMC Womens Health. 2020 Oct 27;20:241. doi: 10.1186/s12905-020-01105-3 (PMC7590717; doi:10.1186/s12905-020-01105-3)
Supplement: Supplementary file 1 — Additional file 1. Pelvic Organ Prolapse and Uterine Preservation: A survey of female gynecologists (POP-UP survey). [file 12905_2020_1105_MOESM1_ESM.pdf]

Pelvic Organ Prolapse and Uterine Preservation: A survey of female gynecologists (POP-UP survey)

English translation of survey questions:

Questionnaire

I. Country of practice? ……………………………………………………………………

II. Type of hospital you are affiliated to?

A University hospital /academic type

B Teaching hospital

C District hospital

D Regional / local hospital

III. What is your age ……. years

IV. What is your area of special interest /subspecialization within OB/GYN

A materno – fetal medicine or antenatal care or obsterical ultrasound

B oncogynaecology

C reproductive medicine

D urogynaecology

E gynaecological endoscopy

F gynaecological endocrinology

G juvenile and adolescent gynaecology

H gynaecological sexuology

I other. Please specify: ………………………………………

J I have no subspecialty (e.g. working in a small hospital, …)

V. Have you completed your family

A Not at all

B Partially, I still plan to have a baby in the future

C I probably have, but cannot say definitely

D I definitely have

Please, answer the following questions based on this scenario.

You are a health postmenopausal woman without any prior major gynaecological surgery. You are suffering from a significant pelvic organ prolapse involving all compartments (anterior, apical, posterior).

VI. What is the likelihood that you would use each of the following options to help you decide the best treatment for you:

	Definitely	Likely	Not likely	Not at all
a urogynecologist				
an oncogynecologist				
another collegaue				
your life partner				
your female friend				
professional literature				
internet, TV, media.				

VII. What is the likelihood that you would opt for each of these options as a management for your POP.

	Definitely	Likely	Not likely	Not at all
No treatment				
Physiotherapy				
Pesary				
Vaginal wall repair				
Sacrospinous fixation				
High uterosacral ligament suspension				
Transvaginal mesh				
Sacrocolpopexy				
Colpocleisis				
Manchester repair				

VIII. Do you believe that uterus is important for your sense of self?

Definitely	Likely	Not likely	Not at all

IX. Regarding uterine sparing or concomitant hysterectomy at the time of POP surgery, what is the likelihood that you would choose the following in case all surgical options were equally effective

	Definitely	Likely	Not likely	Not at all
Total hysterectomy				
Supracervical hysterectomy				
Uterus sparing surgery				

X. If you knew that uterine sparing surgery might have a slightly inferior anatomical outcome, what is the likelihood that you would choose the following

	Definitely	Likely	Not likely	Not at all
Total hysterectomy				
Supracervical hysterectomy				
Uterus sparing surgery				

XI. In your opinion, would a concomitant hysterectomy have a negative impact on:

	Definitely	Likely	Not likely	Not at all
Sense of femininity				
Body image				
Body weight				
Vaginal moisture/lubrication				
Sex drive				
Partner's sex drive				
Partner's sexual satisfaction				

XII. What degree of importance will the following factors have on your decision to have or refuse hysterectomy at the time of POP surgery

	Very important	Important	Not important
Professional's opinion			
Partner's opinion			
Female family member opinion			
Wanting to keep all healthy organs			
Desire to end menstrual periods			
Eliminating need for contraception			

XII. How important are the following factors for you when making a decision about agreeing to or refusing a hysterectomy at the time of POP surgery [2]

	Very important	Important	Not important
Risk of surgical complications			
Pain after surgery			
Mood/emotional state			
Fertility			
Femininity			
Sex drive			
Sexual satisfaction			
Orgasm			
Vaginal lubrication			
Relationship			
Partner's perception			
Partner's sex drive			
Partner's sexual satisfaction			

XIV. Here are some lifetime cancer risks in a woman's life span:

- 1 in 41 women will be diagnosed with uterine cancer during their lifetime
- 1 in 135 women will be diagnosed with cervical cancer during their lifetime.
- 1 in 8 women will be diagnosed with breast cancer during their lifetime
- 1 in 19 women will be diagnosed with bowel cancer during their lifetime
- 1 in 110 women will be diagnosed with bladder cancer during their lifetime
- 1 in 135 women will be diagnosed with stomach cancer during their lifetime

Knowing these risks and knowing the potential of screening, initial signs of cervical and uterine cancer altogether with its curability, please answer the following questions:

XIVa. Regarding uterine sparing or concomitant hysterectomy at the time of POP surgery, what is the likelihood that you would choose the following in case all surgical options were equally effective

	Definitely	Likely	Not likely	Not at all
Total hysterectomy				
Supracervical hysterectomy				
Uterus sparing surgery				

XIVb. If you knew that uterine sparing surgery might have a slightly inferior anatomical outcome, what is the likelihood that you would choose the following

	Definitely	Likely	Not likely	Not at all
Total hysterectomy				
Supracervical hysterectomy				
Uterus sparing surgery				

Bibliography:

1. Korbly NB, Kassis NC, Good MM, Richardson ML, Book NM, Yip S, Saguan D, Gross C, Evans J, Lopes VV, Harvie HS, Sung VW. Patient preferences for uterine preservation and hysterectomy in women with pelvic organ prolapse. Am J Obstet Gynecol. 2013 Nov;209(5):470.e1-6. doi: 10.1016/j.ajog.2013.08.003.
2. Frick AC, Barber MD, Paraiso MF, Ridgeway B, Jelovsek JE, Walters MD. Attitudes toward hysterectomy in women undergoing evaluation for uterovaginal prolapse. Female Pelvic Med Reconstr Surg. 2013 Mar-Apr;19(2):103-9. doi: 10.1097/SPV.0b013e31827d8667.
3. http://www.cancerresearchuk.org
